# Intestinal microbiota link lymphopenia to murine autoimmunity via PD-1^+^CXCR5^−/dim^ B-helper T cell induction

**DOI:** 10.1038/srep46037

**Published:** 2017-04-26

**Authors:** Toshiki Eri, Kimito Kawahata, Takeyuki Kanzaki, Mitsuru Imamura, Kazuya Michishita, Lisa Akahira, Ei Bannai, Noritada Yoshikawa, Yasumasa Kimura, Takeshi Satoh, Satoshi Uematsu, Hirotoshi Tanaka, Kazuhiko Yamamoto

**Affiliations:** 1 Department of Allergy and Rheumatology, Graduate School of Medicine, The University of Tokyo, Tokyo, Japan; 2 Department of Rheumatology and Allergy, IMSUT Hospital, The Institute of Medical Science, The University of Tokyo, Tokyo, Japan; 3 Department of Rheumatology, Graduate School of Medical and Dental Sciences, Tokyo Medical and Dental University (TMDU), Tokyo, Japan; 4 Department of Internal Medicine, Yamanashi Prefectural Central Hospital, Yamanashi, Japan; 5 Division of Systems Immunology, The Institute of Medical Science, The University of Tokyo, Tokyo, Japan; 6 Department of Mucosal Immunology, School of Medicine, Chiba University, Chiba, Japan; 7 Division of Innate Immune Regulation, International Research and Development Center for Mucosal Vaccine, The Institute of Medical Science, The University of Tokyo, Tokyo, Japan; 8 Division of Rheumatology, Center for Antibody and Vaccine Therapy, IMSUT hospital, The Institute of Medical Science, The University of Tokyo, Tokyo, Japan

## Abstract

T cell lymphopenia results in peripheral homeostatic expansion to maintain the T cell immune system, which is termed lymphopenia-induced proliferation (LIP). LIP is a potential risk for expanding autoreactive clones to become pathogenic in human and murine autoimmune diseases. However, the ontogeny of T cells that induce autoantibody production by autoreactive B cells in LIP remains unclear. Transfer of CD4^+^CD25^−^ conventional T (Tc) cells into T-cell-deficient athymic nude mice has been previously reported as a LIP-induced autoimmune model which develops organ-specific autoimmune diseases and systemic antinuclear antibodies (ANAs). We show here that via LIP in this model, Tc cells proliferated and differentiated into PD-1^+^CXCR5^−/dim^ B-helper T cells, which promoted splenic germinal center (GC) formation, provided help for autoantibody-producing B cells, and had distinctive features of follicular helper T (Tfh) cells except that they do not express high CXCR5. Intestinal microbiota were essential for their generation, since depletion of them in recipient mice by antibiotics resulted in a reduction of LIP-induced PD-1^+^CXCR5^−/dim^ B-helper T cells and an amelioration of autoimmune responses. Our findings will contribute to the elucidation of the mechanism of lymphopenia-induced autoimmunity and autoantibody production, and will pave the way for microbiota-targeted novel therapeutic approaches to systemic autoimmune diseases.

Systemic autoimmune diseases are thought to be caused by aberrant activation of self-reactive T and B cells that escape from self-tolerance. It is known that ANAs and other systemic autoantibodies are broadly observed in several human systemic autoimmune diseases such as systemic lupus erythematosus (SLE), Sjogren’s syndrome (SS), and mixed connective tissue disease (MCTD)^1^. However, the ontogeny of self-reactive T and B cells, the mechanisms by which ANA-producing B cells are stimulated or regulated by T cells remain unclear.

Paradoxically, autoimmunity and immunodeficiency can coexist in an individual. For instance, lymphopenia is a clinical feature of systemic autoimmune diseases such as SLE, SS and MCTD^2^. On the other hand, patients with immunodeficiency, such as common variable immunodeficiency^3^ and HIV-infection^4^, have been reported to develop autoimmune diseases or systemic autoimmunity-like conditions. Although the mechanisms of these paradoxes are only partly understood, they can be explained with lymphopenia-induced proliferation (LIP). LIP, also known as homeostatic proliferation, is a physiological peripheral expansion of lymphocytes during lymphopenia, which occurs, for example, during neonatal period, viral infection, and decrease of thymic function in the elderly, in order to reconstitute the immune system and maintain immune homeostasis^5^,^6^. LIP is classified as either “homeostatic” or “spontaneous”, according to the proliferation rate^7^. Homeostatic LIP is relatively slow and dependent on interleukin (IL)-7, whereas spontaneous LIP is rapid, independent of IL-7 and perceived to be driven by T cell receptor (TCR) signal stimulated by self- or commensal bacterial antigens^7^,^8^. Since naïve T cells undergoing robust LIP, can get activated and acquire function as effector/memory T cells^5^,^9^, LIP of T cells has the potential risk of oligoclonal expansion of autoreactive T cells, which are silent until LIP, to be activated to trigger autoimmunity^10^,^11^.

Indeed, LIP is reported to be involved in the pathogenesis of human autoimmune diseases such as SLE^12^, rheumatoid arthritis^6^, and multiple sclerosis^13^, and has been revealed as a direct cause of type-1 diabetes in non-obese diabetes (NOD) mice^14^ and arthritis in K/BxN mice^15^. A classical manipulative LIP-induced autoimmune murine model is neonatal thymectomized mice, which develop multiple organ-specific inflammations including gastritis, thyroiditis, oophoritis, sialoadenitis, and nephritis, with the production of organ-specific antibodies, such as anti-parietal cell antibody^16^,^17^. Sakaguchi *et al*. showed that adoptive transfer of CD4^+^CD25^−^ conventional T (Tc) cells into T cell-deficient athymic nude mice produced similar autoimmune diseases as well as organ-specific and systemic antinuclear antibodies, which were suppressed by CD4^+^CD25^+^ regulatory T (Treg) cells^18^. Inflammatory bowel diseases are also shown to be induced by transfer of naïve CD4^+^ T cells into SCID, RAG^−/−^ or nude mice^19^,^20^,^21^. Although the involvement of interferon-γ or IL-17-producing CD4^+^ T cells in the pathogenesis of these autoimmune diseases has been suggested^22^, the mechanisms that induce and regulate production of organ-specific and systemic autoantibodies in LIP-induced autoimmune diseases have yet to be revealed.

Most pathogenic autoantibodies in human and murine systemic autoimmune diseases are IgG-type and have a high affinity^23^,^24^. This indicates that autoantibody-producing B cells undergo class switching and somatic hypermutation with affinity maturation in germinal centers (GCs)^24^. Follicular helper T (Tfh) cells are a recently identified CD4^+^ T cell subset, which are located within B cell follicles in secondary lymphoid organs and are key regulators of GC reactions and B cell maturation with expression of the distinct transcription factor Bcl-6, surface expression of PD-1, ICOS, CXCR5, and CD200, and production of IL-21^25^,^26^,^27^,^28^. Recent studies have shown that Tfh cells have pathogenic roles in autoantibody production in several murine lupus models via interaction with B cells with ICOS and IL-21^29^,^30^,^31^,^32^,^33^,^34^. An increased number of circulating Tfh cells is also reported in human lupus patients^35^,^36^.

Intestinal microbiota have been shown to be essential in “spontaneous” LIP, since immunodeficient mice raised under germ-free conditions failed to cause spontaneous LIP^8^. The roles of intestinal microbiota in the maintenance of immune homeostasis and their association with autoimmune diseases have been under intense study in recent years. Raised in germ-free or intestinal microbiota-depleted condition, K/BxN mice significantly ameliorated arthritis^37^, however, NOD mice showed higher incidence of Type 1 diabetes^38^,^39^,^40^. These findings imply that intestinal microbiota can be proinflammatory or suppressive, depending on the host strains and diseases. However, since germ-free-grown classical lupus model mice, such as NZB, MRL/lpr, and the pristane-injected, showed no significant differences in disease activity^41^, the roles of microbiota in these systemic autoimmune mice with the production of ANAs have not yet been elucidated.

Until now, autoimmune disease, lymphopenia, and commensal bacteria have been shown to be linked but their detailed relationship is only partially understood. Here we show that in LIP-induced autoimmunity, Tc cells proliferate and differentiate into Tfh cells under the presence of intestinal microbiota, which promote GC formation and help autoreactive B cells to produce autoantibodies. Furthermore, antibiotic treatment to deplete intestinal microbiota inhibited the generation of LIP-induced Tfh cells and the subsequent development of autoantibodies and autoimmune diseases.

## Results

### Immunoglobulin production, class switching, and antinuclear antibody production induced by LIP of CD4^+^CD25^−^ T cells

**Figure 1:**
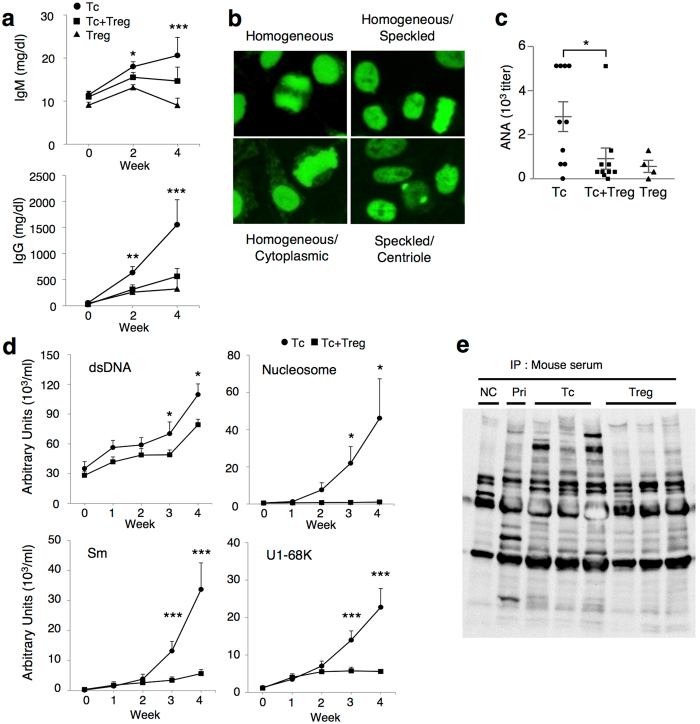
CD4^+^CD25^−^ Tc cell-transferred nude mice produce IgG and various antinuclear antibodies. Tc cells and Treg cells were isolated from the splenocytes of BALB/c WT mice and transferred separately or together into BALB/c *nu/nu* mice. Tc, n = 10, Tc + Treg, n = 10, Treg, n = 4. (**a**) Total IgM and IgG concentrations in sera from recipient *nu/nu* mice were determined by ELISA. (**b**) ANAs in the recipient sera 3 weeks after Tc cell-transfer were detected by immunofluorescence assay. Representative patterns of cellular staining of HEp-2 cells by sera diluted at 1:40 followed by development with Alexa 488-conjugated goat anti-mouse IgG are shown. Original magnification x400. (**c**) Titers of ANAs in the sera from each recipient 4 weeks after the transfer. (**d**) Various ANAs detected by ELISA within 4 weeks after the transfer. (**e**) Immunoprecipitation of serum antibodies to nuclear antigens. Extracts of nuclear components of spleens from WT mice were biotinylated and reacted with sera taken from WT normal control mice (NC), pristane-induced lupus model mice (Pri), or *nu/nu* mice 4 weeks after the transfer of Tc or Treg cells, followed by immunoprecipitation with mAbs to mouse IgG. Cumulative plot data and bars are shown as mean ± SEM from 2 independent experiments. *P < 0.05, **P < 0.01, ***P < 0.005.

In a previous study, CD4^+^CD25^−^ Tc cell-transferred nude mice developed multiple organ-specific autoimmune diseases and produced ANAs and anti-parietal cell antibodies, which were suppressed by CD4^+^CD25^+^ Treg cells^18^. We then focused on the mechanism of antibody production in this T cell-transferred LIP murine model. CD4^+^CD25^−^ Tc cells and CD4^+^CD25^+^ Treg cells from BALB/c wild-type (WT) mice were adoptively transferred separately or together into T cell-deficient BALB/c nude (*nu/nu*) mice which lack thymus. Within 4 weeks, Tc cell-transferred *nu/nu* recipients developed significantly increased production of IgM and IgG, suggesting class switching of B cells (Fig. 1a). Co-transfer of Treg cells suppressed them (Fig. 1a). Immunofluorescence microscopy revealed production of various patterns of IgG-type ANAs in the serum of the Tc cell-recipients, especially a homogeneous pattern was dominant (Fig. 1b). The Tc cell-recipients produced ANAs with a significantly higher titer at a higher positive ratio, almost 100%, within 4 weeks (Fig. 1c). The production of ANAs was suppressed when Treg cells were co-transferred, and not induced when only Treg cells were transferred (Fig. 1c). Antibodies against specific nuclear antigens, such as double-stranded DNA (dsDNA), nucleosome, Sm, and U1-68K, which are known to be observed in human systemic autoimmune diseases, were also elevated in the Tc cell-recipients and suppressed by Treg cells (Fig. 1d). Immunoprecipitation of nuclear extracts in the sera confirmed that antibodies recognizing various nuclear self-antigens were produced in Tc cell-recipients (Fig. 1e). These findings indicate that LIP of Tc cells transferred into T cell-deficient recipients promotes class switching of B cells and breaks B cell tolerance, resulting in ANA production, and that Treg cells inhibit aberrant B cell response during LIP.

### Germinal center formation and generation of CD4^+^PD-1^+^ICOS^+^CD200^+^CXCR5^−/dim^ cells after the LIP of transferred Tc cells

**Figure 2:**
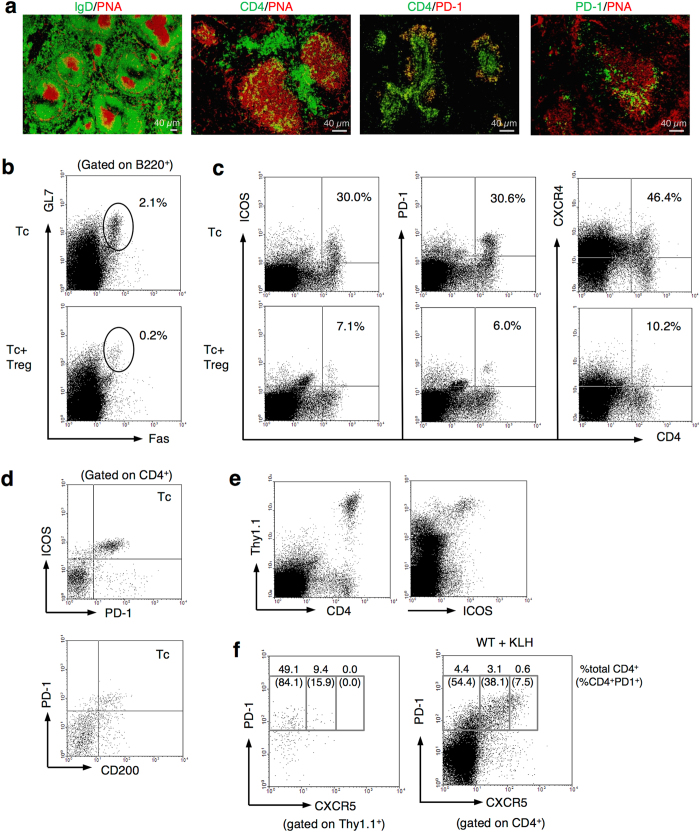
LIP of Tc cells generates germinal centers and ICOS^+^PD-1^+^CD200^+^CXCR4^+^CXCR5^−/dim^ CD4^+^ T cells. (**a**) Immunofluorescence staining in spleens from recipient *nu/nu* mice 5 days after Tc cell-transfer. Sections were stained with PNA (red) and antibodies against IgD (green), CD4 (green), and PD-1 (red and green). (**b–d**) Flow cytometry analysis of splenocytes from recipient *nu/nu* mice 5 days after Tc cell- or Tc and Treg cell-transfer. Representative data plot and cumulative data indicate percentage of GL7^+^Fas^+^ on B220^+^ cells (**b**), and ICOS^+^, PD-1^+^, CXCR4^+^ on CD4^+^ cells (**c**). (**d**) Co-staining of PD-1, ICOS and CD200 on CD4^+^ T cells after Tc cell-transfer. (**e,f**) Tc cells from Thy1.1^+^ WT mice were transferred into Thy1.2^+^ *nu/nu* mice. Splenocytes from the recipient mice were stained with Thy1.1, CD4, ICOS, and CXCR5, and analyzed by flow cytometry. Lymph nodes from WT mice 7 days after the immunization with KLH (WT   +KLH) are shown as positive controls of CXCR5^+^ Tfh cells in (**f**). Data are representative of 2 independent experiments with 3-to-4 mice per group.

Class-switched antibodies observed in Tc cell-recipients suggested the interaction of B cells with B-helper T cells in GC. Histological analysis of the spleen from *nu/nu* recipient mice 5 days after Tc cell-transfer revealed the formation of GCs stained with peanut-agglutinin (PNA) surrounded by IgD^+^ B cell follicles (Fig. 2a). CD4^+^ cells were distributed in the T cell zone and GCs, and especially CD4^+^ cells localizing in GC expressed PD-1, one of the surface markers of Tfh cells (Fig. 2a). Tc cells before transfer contained very few PD-1^+^ICOS^+^CD4^+^ cells (see Supplementary Fig. S1). Then, to examine whether transferred Tc cells differentiated into Tfh cells and formed GC with B cells, we analyzed the phenotype of B and T cells in the spleens of recipient *nu/nu* mice 5 days after T by flow cytometry. As expected, GC-B cells which is recognized as GL7^+^Fas^+^B220^+^ cells^42^,^43^ and CD4^+^ T cells expressing Tfh surface markers PD-1, ICOS, and CD200^25^ were generated after Tc cell-transfer (Fig. 2b–d). CXCR4, which is also known to be expressed by B-helper T cells^29^, was also increasingly expressed after Tc cell-transfer (Fig. 2c). Their generation was suppressed by co-transfer of Treg cells (Fig. 2b,c).

**Figure 3:**
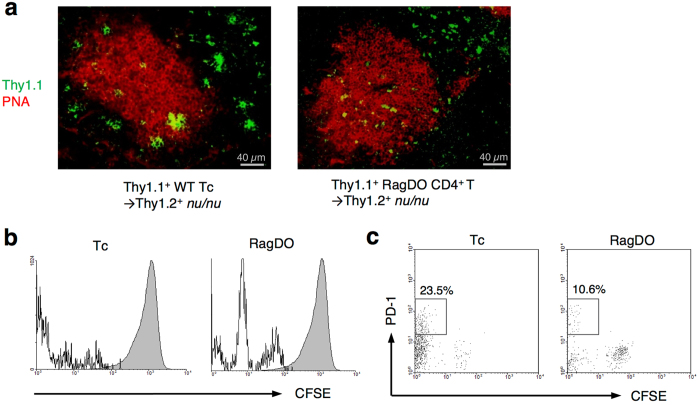
Rapidly divided Tc cells differentiate into PD-1^+^CD4^+^ T cells and localize in germinal centers, regardless of TCR specificity. (**a**) Tc cells from Thy1.1^+^ WT mice or CD4^+^ T cells from Thy1.1^+^ Rag2^−/−^DO11.10 mice (RagDO) were transferred into Thy1.2^+^ *nu/nu* mice. Cryosections of spleens of the recipient mice 5 days after the transfer were stained with anti-Thy1.1 antibody (green) and PNA (red). Bars; 40 μm. (**b**,**c**) Tc cells or CD4^+^ T cells from RagDO mice were stained with CFSE and transferred into *nu/nu* mice. Splenocytes from the recipient mice were analyzed by flow cytometry after 5 days. (**b**) Detection of division of the transferred CFSE-labeled CD4^+^ cells. (**c**) PD-1 expression on rapidly divided CD4^+^ cells.

It is known that *nu/nu* mice have intrinsic extrathymically developed CD4^+^ T cells^44^. To confirm that the splenic PD-1^+^ICOS^+^CD200^+^CD4^+^ cells did not originate from recipient *nu/nu* mice but from adoptively transferred Tc cells, Tc cells from Thy1.1^+^ WT mice were transferred into Thy1.2^+^ *nu/nu* mice. Transferred Thy1.1^+^ Tc cells indeed turned out to differentiate into PD-1^+^ICOS^+^ cells (Fig. 2e). Interestingly, surface expression of CXCR5, which is perceived to be a distinctive molecule of traditional Tfh cells, in the proliferated CD4^+^ cells was not as high as typical Tfh cells induced by Keyhole limpet hemocyanin (KLH)-immunization of WT mice (Fig. 2f), though they localized in the GCs (Fig. 3a, left). Carboxyfluorescein succinimidyl ester (CFSE)-staining of Tc cells revealed that these T cells were differentiated from rapidly proliferated cells, which is a characteristic of “spontaneous” LIP^7^ (Fig. 3b,c, left).

These results showed that CD4^+^CD25^−^ Tc cells transferred into lymphopenic recipients proliferated and differentiated into PD-1^+^ICOS^+^CD200^+^CXCR5^−/dim^CD4^+^ T cells via spontaneous LIP, and were involved in the formation of GCs with B cells. The generation of PD-1^+^ICOS^+^CD200^+^CXCR5^−/dim^CD4^+^ T cells and GC-B cells were suppressed by Treg cells.

### LIP-induced PD-1^+^ICOS^+^CD200^+^CXCR5^−/dim^CD4^+^ T cells function as Tfh cells

Because these LIP-induced T cells in our experiments did not express high CXCR5, we next investigated whether the LIP-induced PD-1^+^ICOS^+^CD200^+^CD4^+^ T cells actually function as Tfh cells; that is, formation of GCs, interaction with B cells with expression of the transcriptional factor Bcl-6^45^, and production of the cytokine IL-21^25^.

**Figure 4:**
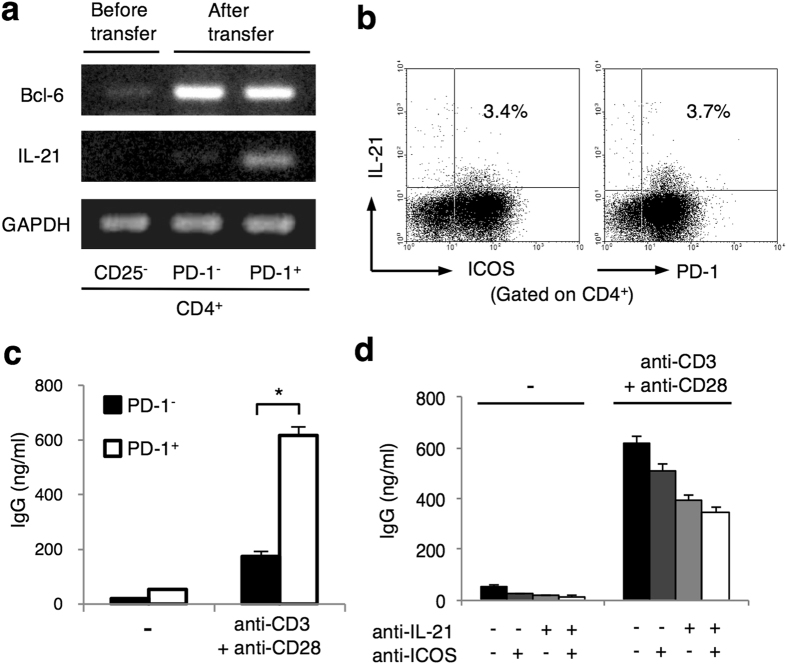
LIP-induced PD-1^+^CD4^+^ T cells function as follicular helper T cells. CD4^+^ T cells were isolated from spleens of recipient *nu/nu* mice 5 days after Tc cell-transfer. Isolated CD4^+^ T cells were further separated into PD-1^+^ or PD-1^−^ cells in (**a**), (**b**) and (**d**). (**a**) RT-PCR analysis of *Bcl-6* and *Il-21* mRNA expression in Tc cells before the transfer, and CD4^+^PD-1^−^ cells and CD4^+^PD-1^+^ cells after the transfer. (**b**) Intracellular staining of IL-21 in LIP-induced ICOS^+^PD-1^+^ CD4^+^ T cells from the spleens of recipient *nu/nu* mice. Numbers in quadrants represent the percentage. Data are representative of 2 independent experiments. (**c** and **d**) CD19^+^ B cells from WT mice were co-cultured with CD4^+^PD-1^+^ cells (**c** and **d**) or CD4^+^PD-1^−^ cells (**c**) separated after the transfer. Cells were stimulated with anti-CD3 and anti-CD28 antibodies (**c** and **d**) and with neutralizing antibodies to IL-21 (10 μg/ml) and/or ICOS (10 μg/ml) (**d**). IgG production in the supernatant was measured by ELISA (mean ± SEM). Data are representative of 2 independent experiments with 3 mice. *P < 0.05.

RT-PCR revealed mRNA expression of *Bcl-6* and *Il-21* in PD-1^+^CD4^+^ T cells generated after Tc cell-transfer (Fig. 4a). Intracellular IL-21 expression in the LIP-induced PD-1^+^ICOS^+^CD4^+^ T cells was also confirmed by flow cytometry (Fig. 4b). To investigate the ability to help B cells produce antibodies, LIP-induced PD-1^+^CD4^+^ T cells or PD-1^−^CD4^+^ T cells were co-cultured with B cells under CD3/CD28 stimulation. LIP-induced PD-1^+^CD4^+^ T cells showed a significantly greater ability to promote IgG production (Fig. 4c). Additionally, neutralization of ICOS or IL-21 inhibited the capacity to promote IgG production in an additive manner, although not completely. These findings reveal that LIP-induced PD-1^+^ICOS^+^CD200^+^CXCR5^−/dim^ CD4^+^ T cells indeed have the function of Tfh cells, in that they express Bcl-6, produce IL-21, and have the capacity to promote IgG production by B cells via ICOS and IL-21. Accordingly, we labeled these LIP-induced PD-1^+^ICOS^+^CXCR5^−/dim^ B-helper T cells as “LIP-Tfh cells”.

### Contribution of T cell receptor specificity to the ANA production and organ-specific autoimmune diseases

**Figure 5:**
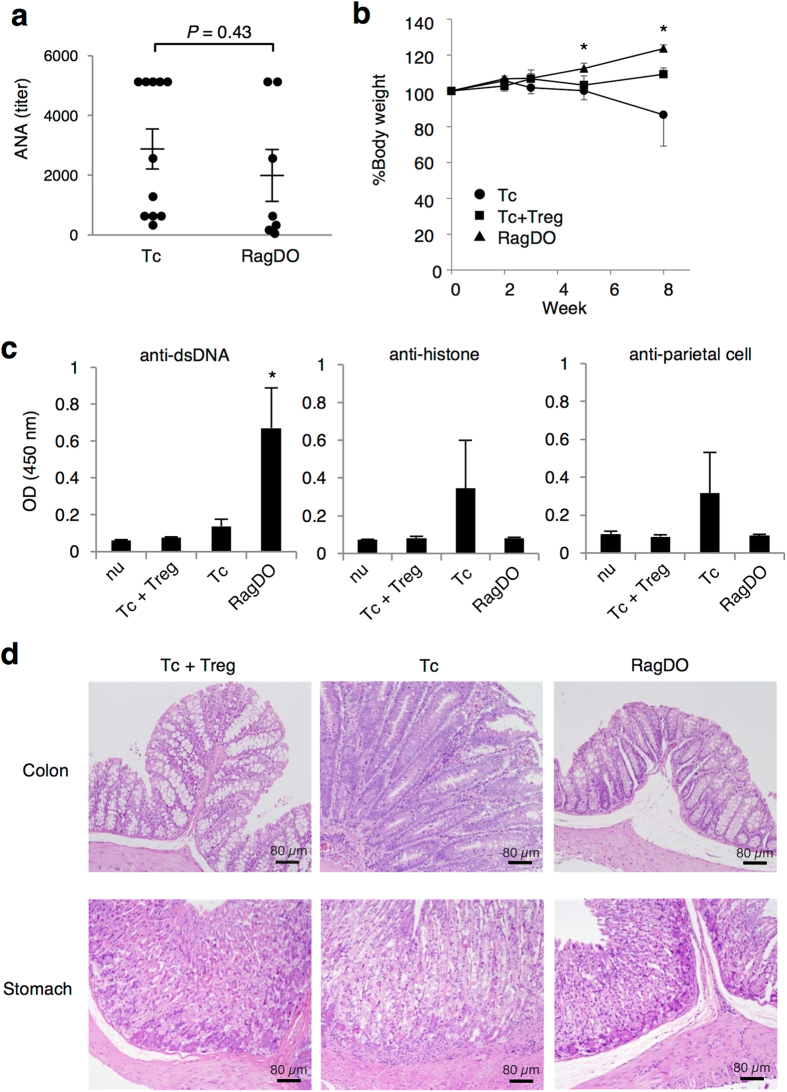
T cell specificity contributes not to systemic autoantibodies, but to organ-specific autoimmunity. Tc cells, Tc and Treg cells, or CD4^+^ T cells from Rag2^−/−^DO11.10 mice (RagDO) were transferred into *nu/nu* mice. (**a**) ANA titer in the sera from recipients was detected by immunofluorescence assay 3 weeks after the transfer. Each dot represents an individual mouse and bars represent the mean ± SEM. (**b**) Weight changes of the recipient mice. Data are from 2 independent experiments with 5 mice per group (mean ± SEM). *P < 0.05. (**c**) Anti-dsDNA, anti-histone, and anti-parietal antibodies in the serum of control *nu/nu* mouse (nu) or each recipient mouse 4 weeks after the transfer were detected by ELISA. Data are from 5 mice per group (mean ± SEM). *P < 0.05. (**d**) Hematoxylin and eosin staining of the colon and stomach from the recipient mice 8 weeks after the transfer. Representative image of 3 mice per group.

To further investigate the ontogeny of LIP-Tfh cells, we analyzed the contribution of T cell receptor (TCR) specificity to the differentiation and function of LIP-Tfh cells using TCR-restricted T cells. Previously, our group reported that Rag2^−/−^ DO11.10 mice (RagDO), which have a unique TCR specific for ovalbumin (OVA) in all CD4^+^ T cells, do not have any naturally occurring regulatory T (nTreg) cells in the periphery^46^. When RagDO-derived CD4^+^ T cells were transferred into *nu/nu* mice, cell proliferation, differentiation into LIP-Tfh cells, and localization of these cells into GCs were observed. (Fig. 3a–c). Some RagDO-CD4^+^ cells showed slower proliferation (indicative of “homeostatic” proliferation) than WT Tc cells, however, rapidly proliferated cells (indicative of “spontaneous” proliferation) differentiated into PD-1^+^ LIP-Tfh cells as well as WT Tc cells (Fig. 3b,c). Furthermore, ANAs were also produced by RagDO-CD4^+^ T cell-transferred recipients to the same degree as Tc cell-transferred mice (Fig. 5a). These results imply that TCR specificity of proliferating cells is not involved in LIP-Tfh cell differentiation to promote ANA production. On the other hand, RagDO-CD4^+^ T cell-transferred *nu/nu* mice did not show wasting disease, which was observed in Tc cell-transferred *nu/nu* mice at 8 weeks of age (Fig. 5b). Anti-parietal cell antibody, which is associated with the pathogenesis of autoimmune gastritis^17^, was induced by WT-Tc cell-transfer but not by RagDO-CD4^+^ T cell-transfer (Fig. 5c). Histological analysis revealed development of colitis (cell infiltration, loss of goblet cells, mucin depletion, crypt elongation, and crypt abscesses) and gastritis (cell infiltration, oxyntic atrophy, and hyperplasia/metaplasia) in Tc cell-transferred *nu/nu* mice, as with previous reports (Fig. 5d). However, RagDO-CD4^+^ T cell-transferred *nu/nu* mice showed almost no colitis or gastritis, though slight cell infiltration in stomach mucosa was observed (Fig. 5d). ELISA analysis of various ANAs revealed the RagDO-CD4^+^ T cell-transfer promotes the production of anti-dsDNA and anti-Scl70 antibodies, but did not promote antibodies against histone, RNP/Sm, RNP-70, SS-A, and SS-B that were produced by transfer of WT Tc cells (Fig. 5c, and data not shown). These findings indicate that TCR specificity of proliferating cell is not involved in the induction of LIP, the generation of LIP-Tfh cells, and the production of ANAs, but involved in the development of organ-specific autoimmune diseases with autoantibodies and the diversity of ANAs. We also confirmed that co-transfer of Treg cells suppressed all these antibodies and autoimmune diseases promoted by WT Tc transfer (Fig. 5c,d).

### High-affinity interaction of TCR with intranuclear antigen suppresses LIP-Tfh cell generation

**Figure 6:**
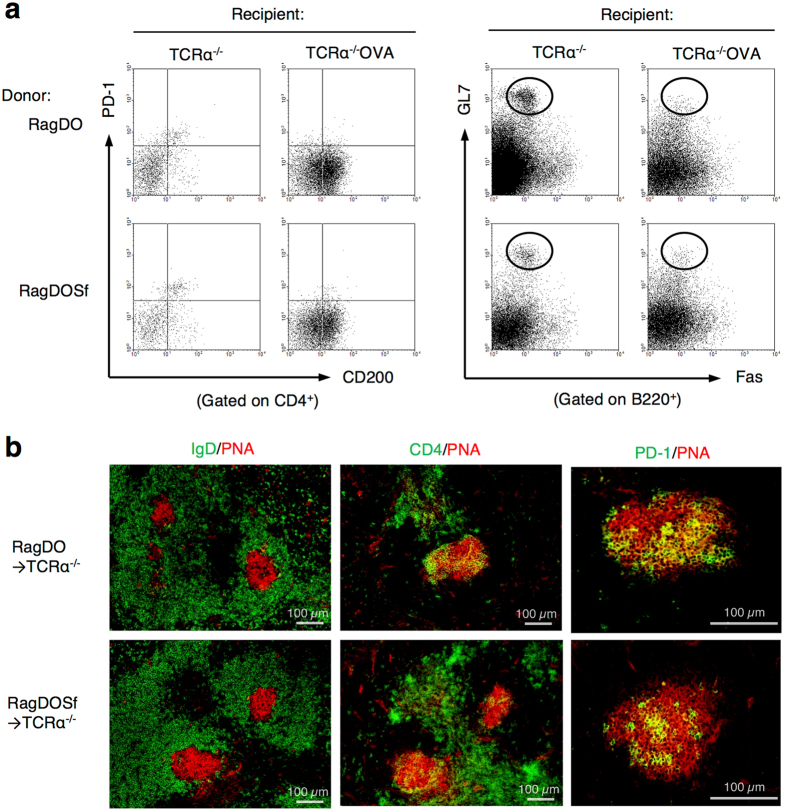
CD4^+^ T cells with unique TCR were transferred into T cell-deficient recipients bearing cognate intranuclear antigens. CD4^+^ cells from Rag2^−/−^DO11.10 mice (RagDO) or RagDO scurfy mice (RagDOSf) were transferred into TCRα^−/−^ or TCRα^−/−^ Ld-nOVA recipient *nu/nu* mice and spleens were analyzed after 5 days. (**a**) Flow cytometry of PD-1^+^CD200^+^CD4^+^ T cells and B220^+^GL7^+^Fas^+^ GC-B cells in the splenocytes. (**b**) Immunofluorescence of spleens from recipient TCRα^−/−^ mice 5 days after the transfer. Cryosections were stained with PNA (red), and antibodies against IgD (green, left), CD4 (green, middle), and PD-1 (green, right). Representative images from 2 independent experiments.

To further investigate the involvement of cognate antigen stimulation of TCR, we used Ld-nOVA transgenic mice, which express OVA in all the cell nuclei^46^. We observed that Tc cell-transfer into another strain of T cell-deficient mice, TCR alpha-chain^−/−^ (TCRα^−/−^) mice or Ld-nOVA TCRα^−/−^mice, induced LIP, generation of LIP-Tfh cells, formation of GCs, and production of ANAs, as well as Tc cell-transfer into *nu/nu* mice (data not shown). Then, transfer of RagDO-CD4^+^ T cells into TCRα^−/−^ mice induced generation of PD-1^+^ LIP-Tfh cells which localize in GC, and GC-B cells as well (Fig. 6a,b). However, transfer of RagDO-CD4^+^ T cells into Ld-nOVA-TCRα^−/−^ mice produced significantly decreased generation of PD-1^+^ LIP-Tfh and GC-B cells (Fig. 6a). These findings imply that generation of LIP-Tfh cells promoting ANA production does not need the TCR recognition of nuclear antigen, but rather high-affinity TCR interaction with self-antigen instead impair the generation of LIP-Tfh and GC-B cells.

Furthermore, since Foxp3^+^CD4^+^ Treg cells have been reported to differentiate into Tfh cells, so-called follicular regulatory T cells, which localize in GCs and suppress GC reaction^47^, we examined the possibility of whether Foxp3^+^CD4^+^ T cells, of which there are few if any, were the origin of LIP-Tfh cells. When we transferred CD4^+^ T cells from scurfy-mutated, i.e. lacking in *foxp3*, RagDO mice into TCRα^−/−^ or Ld-nOVA-TCRα^−/−^ mice, the generation of LIP-Tfh cells and GC-B cells and the localization of Tfh cells in the GCs were not affected (Fig. 6a bottom side and 6b bottom side), indicating that LIP-induced Tfh cells were not differentiated from Foxp3^+^ Treg cells.

### Involvement of intestinal microbiota in LIP and LIP-Tfh cell generation

IL-7 independent rapid LIP is believed to be induced by self- or commensal bacterial antigen recognition, and past study using germ-free mice showed significantly impaired rapid “spontaneous” LIP^8^. We therefore investigated the roles of intestinal microbiota in the generation of LIP-Tfh cells and the development of autoimmune diseases in our LIP-induced autoimmune murine model.

**Figure 7:**
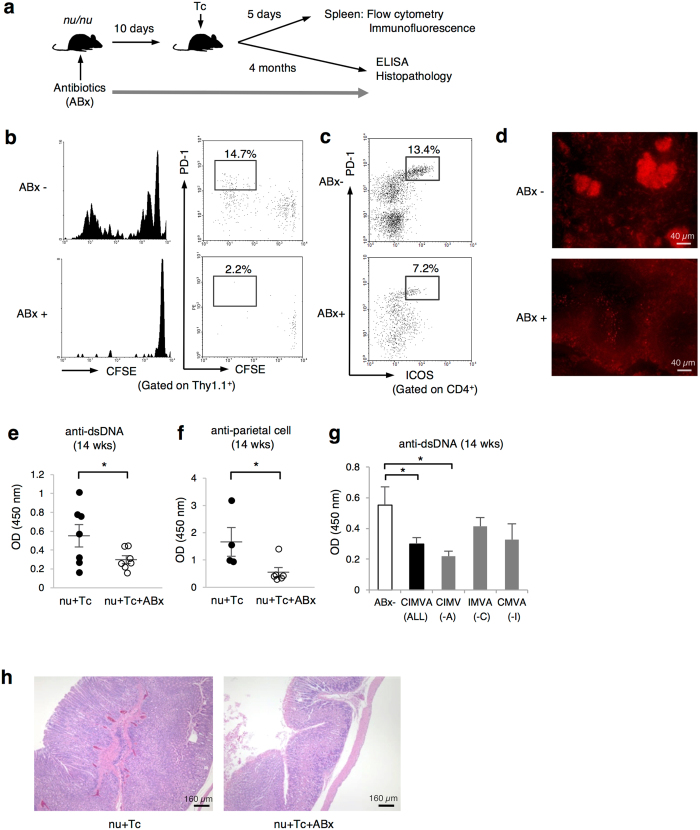
Depletion of intestinal microbiota shows their essential roles in LIP, LIP-Tfh cell induction, and germinal center formation. (**a**) Scheme of the experimental approach. Recipient *nu/nu* mice were administered antibiotics composed of ciprofloxacin, imipenem, metronidazole, vancomycin, and amphotericin B in drinking water for 10 days prior to Tc cell-transfer. Spleens of the recipients were analyzed 5 days after the transfer (**b–d**). Autoimmune antibodies and diseases in the recipients were observed for 4 months (**e–h**). Treatment with antibiotics was continued until mice were sacrificed. (**b**) CFSE-labeled Thy1.1^+^ Tc cells were transferred into *nu/nu* mice with (ABx+) or without the antibiotics (ABx−) treatment. CFSE dilution and PD-1 expression in transferred Thy1.1^+^ cells were assessed by flow cytometry. Representative flow cytometry plots percentages of PD-1-positive divided cells are shown. (**c**) Percentage of PD-1^+^ICOS^+^CD4^+^ LIP-Tfh cells were indicated. (**d**) Immunofluorescence of spleens from the recipient mice. Cryosections were stained with PNA. Data are representative of 2 independent experiments with 3 mice per group. (**e** and **f**) Recipient mice were treated with or without the 5 antibiotics for 14 weeks. Anti-dsDNA antibody (**e**) and anti-parietal cell antibody (**f**) in the sera from each mouse were measured by ELISA. Data are from 4-to-7 mice per group, mean ± SEM. *P < 0.05. (**g**) Combinations of all or 4 of the 5 antibiotics were administered to recipient *nu/nu* mice: ciprofloxacin (C), imipenem (I), metronidazole (M), vancomycin (V), and amphotericin B (A). Anti-dsDNA antibody in the sera 14 weeks after Tc cell-transfer was determined by ELISA. Data were from 5 mice per group, mean ± SEM. *P < 0.05. (**h**) Hematoxylin and eosin staining of stomachs from the recipient mice with or without administration of C, I, M, and V at 19 weeks after the transfer.

Intestinal microbiota of the recipient *nu/nu* mice were depleted by oral administration of 5 broad-spectrum antibiotics (Ciprofloxacin: CPFX, Imipenem: IPM, Metronidazole: MDZ, Vancomycin: VCM, and Amphotericin B: AMB) in ad libitum drinking water. Selection of antibiotics was determined on the basis of previous reports^48^. Ten days after beginning antibiotics administration, CFSE-labeled Thy1.1^+^ Tc cells were transferred into *nu/nu* mice, and the spleens were harvested and analyzed 5 days after the transfer (Fig. 7a). In antibiotics-treated recipients, LIP, differentiation into LIP-Tfh cells, and GC formation significantly diminished (Fig. 7b–d), suggesting that intestinal microbiota has essential roles in LIP and LIP-Tfh cell generation. Tfh polarizing cytokines (IL-6 and IL-2) and “homeostatic” proliferation inducing cytokines (IL-7 and IL-15) in spleen were not affected by microbiota depletion (see Supplementary Fig. S2). Proliferated Tc cells acquired alpha 4 beta 7 integrin, a gut homing receptor^49^, and lost CCR7, a lymph node and secondary lymphoid organ homing receptor which is reported to be expressed on naïve T cells and not expressed on effector memory cells and circulating Tfh precursor cells^50^ (see Supplementary Fig. S3). These suggest migration of proliferated Tc cells from spleen and lymph nodes to gut tissue when they are primed to differentiate into Tfh cells. Intriguingly, Bcl-6 expression in transferred Tc cells was not decreased by antibiotic treatment (see Supplementary Fig. S3).

### The depletion of intestinal microbiota with antibiotic treatment ameliorated autoantibodies and autoimmune gastritis

To further investigate the roles of intestinal microbiota in LIP-induced autoimmunity, we observed the effect of long-term antibiotic treatment on autoantibody production and autoimmune diseases. Oral antibiotics to recipient mice as described above were started 5 days before Tc cell-transfer and continued during the observation period of 14 weeks. The production of anti-dsDNA and anti-parietal cell antibodies was significantly suppressed by the depletion of intestinal microbiota (Fig. 7e,f). Next, we examined the effects of different combinations of antibiotics on their suppression of anti-dsDNA antibodies. The combination of 4 antibiotics without AMB also significantly suppressed autoantibody production (Fig. 7g). This suggests that the combination of CPFX, IPM, MDZ, and VCM are sufficient to deplete microorganisms which are involved in LIP-Tfh cell-associated autoantibody production. After 19 weeks with CPFX, IPM, MDZ, and VCM treatment, LIP-induced autoimmune gastritis was significantly suppressed (Fig. 7h). These results suggest that intestinal microbiota susceptible to these 4 antibiotics play a critical role in autoantibody production and autoimmune gastritis in this LIP-induced autoimmune murine transfer model over a long period.

**Figure 8:**
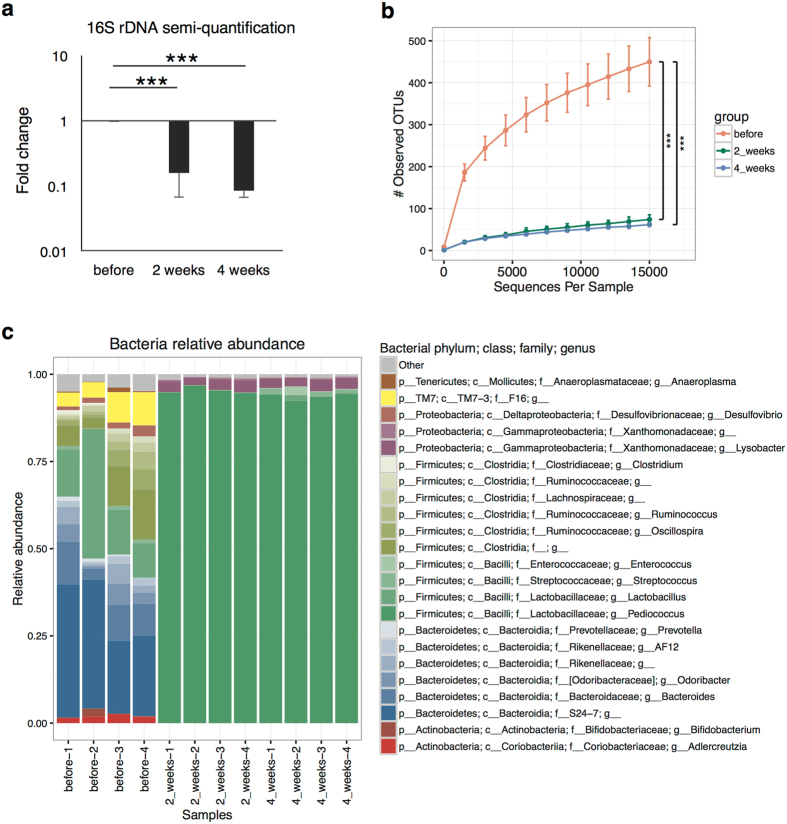
16S rRNA gene analysis in the feces of antibiotic-treated mice. Stool samples were collected from mice before, 2 weeks after, or 4 weeks after antibiotic treatment with ciprofloxacin, imipenem, metronidazole, and vancomycin (n = 4 per each group). **(a)** Semi-quantification of 16 S rDNA gene abundance per stool (gram) by PCR with agarose gel electrophoresis (mean ± SEM); ***P < 0.001. **(b**) Rarefaction curves of observed OTUs based on 16S rDNA gene sequences. *P* values from student’s *t*-test; ***P < 0.001. **(c**) Relative abundance of bacterial genera based on 16S rDNA gene sequences.

Since antibiotic-treatment had significant effects to inhibit the generation of autoantibody-promoting LIP-Tfh cells and ameliorate autoimmune diseases in LIP-autoimmune model, we investigated the change in the intestinal composition of bacterial communities by fecal analysis. DNA from fecal samples were isolated and V3 and V4 region of bacterial 16S rRNA genes were amplified by PCR and the sequencing were performed. We found significant reduction in bacterial 16S rDNA copies and diversities in feces after 2 weeks and 4 weeks of antibiotic treatment (Fig. 8a,b). 16S rDNA sequence data analysis further revealed that almost all the kinds of intestinal bacteria were successfully depleted by the combination of the 4 antibiotics within 2 weeks, and the condition was kept with continual administration for 4 weeks (Fig. 8c). Interestingly, Pediococcus genus in Lactobacilllaceae family, which were reported to have probiotic effects in autoimmune diseases^51^, survived and became dominant after antibiotic treatment, although they were not detected before the treatment (Fig. 8c).

## Discussion

This study has demonstrated that CD4^+^CD25^−^ T cells transferred in syngeneic T cell-lymphopenic recipients proliferate via LIP and differentiate into Tfh cells under the presence of intestinal microbiota. These LIP-induced Tfh cells promote systemic (antinuclear) and organ-specific (anti-parietal cell) autoantibody production by autoreactive B cells. Although the phenomenon that lymphopenic mice can induce several autoimmune diseases, such as autoimmune gastritis, sialoadenitis, oophoritis and thyroiditis, has been documented for many years^17^,^18^, the subset of T cells involved in autoantibody production has not yet been revealed. To our knowledge, this is the first report to reveal that Tfh cell generation promotes systemic and organ-specific autoantibody production in LIP-induced autoimmunity. We also replicated the evidence that CD4^+^CD25^+^ Treg cells suppress aberrant B cell reaction and autoimmune diseases during LIP^18^.

This LIP-induced autoimmune mouse produced IgG-type ANAs with high-affinity, at a high rate within 4 weeks. This is much quicker than in traditional lupus model mice, such as MRL/lpr, NZB/WF1, BXSB-Yaa, or pristane-injected mice, which take several months to begin producing ANAs^52^,^53^. Furthermore, it is complicated that these classical lupus model mice should be kept on special genetic background or injected non-physiological chemical material. In contrast, it is notable that this transfer model is simpler and able to assess ANA production and its regulation within a month. Thus, this model seems more useful as an ANA-producing autoimmune murine model.

It should be noted that our LIP-induced Tfh cells do not express high CXCR5, which is perceived as a distinctive Tfh surface molecule. CXCR5 is a transmembrane receptor for chemokine CXCL13 and its interaction with CXCL13 expressed in B cell-follicles is known to be important for Tfh cells to migrate and form GCs^26^,^27^. However, CXCR5^−/dim^ LIP-Tfh cells in our model could also significantly form and localize in GCs. We then assessed the function of these cells and concluded that CXCR5^−/dim^ LIP-Tfh cells are definitely Tfh cells because of (1) distinctive surface markers; PD-1, ICOS, and CD200, (2) distinctive transcription factor; Bcl-6, (3) cytokine production; IL-21, (4) localization in GCs, and (5) function to help B cell via ICOS and IL-21. Past studies of CXCR5^−/−^ mice have shown that GC formation was impaired, but not totally blocked, implying that CXCR5 expression by Tfh cells is important but not indispensable for GC formation^54^,^55^. Moreover, a homing receptor other than CXCR5 was recently found to be involved in Tfh cell localization in GC^56^. Since some of LIP-Tfh cells express low CXCR5, there is a possibility that LIP-Tfh cells use CXCR5 to localize in GC, however, these reports support the idea that LIP-Tfh cells in our model can localize in GC even if they lack CXCR5. A recent finding that IL-21^+^ICOS^+^CXCR5^−^CD4^+^ T cells expand in the human blood and help antibody production after vaccination also supports the presence of CXCR5^−^ Tfh cells^57^, although whether CXCR5^−^ Tfh cells induced by infection are the same as those induced in autoimmunity remains unclear.

Accumulating data have revealed the pathogenic role of IL-21 produced by Tfh cells or Tfh-like cells in murine and human autoimmune diseases^33^,^34^,^58^. Not only Tfh cells, CXCR5^−^CXCR4^+^ICOS^+^ extrafollicular helper T cells were reported to produce IL-21, provide help to B cells, and be involved in the pathogenesis of traditional autoimmune model mice^32^, and these cells were also Bcl-6-positive^59^. Recent reports showed that IL-21-producing PD-1^+^CXCR5^−^CD4^+^ T cells were localized in inflamed tissue^60^ and interacted with B cells to produce antibodies at that site^61^. Since these data exhibited the possibility that CXCR5^−^ B helper T cells were also extrafollicularly involved in autoantibody production in autoimmunity models, further investigation is needed to fully describe LIP-induced PD-1^+^CXCR5^−/dim^CD4^+^ B-helper T cells in the pathogenesis of systemic autoimmune diseases.

Previous reports classified LIP into two different patterns; slow IL-7 dependent “homeostatic” proliferation and rapid, self- or commensal antigen-dependent “spontaneous” proliferation, and the latter was demonstrated to lead to differentiation into effector T cells and to be associated with autoimmunity, as well as being suppressed by Treg cells^7^,^8^,^11^. Our findings that rapidly proliferated cells differentiated into autoimmunity-related Tfh cells depending on the presence of intestinal microbiota, and that Treg cells suppressed the rapid proliferation and the differentiation, were consistent with those of previous studies. However, the finding that CD4^+^ T cells with OVA-specific TCR, which are assumed not to recognize any self- or commensal antigens in mice, could also proliferate and differentiate into Tfh cells to promote ANA production is not consistent with the features of previously reported “spontaneous” proliferation. The dependence of LIP on intestinal microbiota, but not on TCR specificity, suggests that T cells in LIP recognize bacteria by means other than TCR. Past studies have reported that dendritic cells (DCs) stimulated by bacterial antigen via toll-like receptors (TLR) promote T cell proliferation with IL-6^62^,^63^, implying that TLR recognition of bacteria and subsequent production of cytokines can explain the discrepancy in our model. Since our result showed no significant change in cytokines in the spleen after microbiota depletion, further investigation is needed to validate these hypotheses.

Importantly, non-self-reactive (e.g. OVA-specific) TCR-restricted LIP-Tfh cells promoted ANAs in a shorter time, but did not induce anti-parietal cell antibody, gastritis, colitis, or wasting disease in a longer period. This finding indicates that LIP-induced systemic autoantibodies, such as ANAs, are promoted by LIP-Tfh cells independent of their TCR specificity, but that LIP-induced organ-specific autoimmune diseases are induced by LIP of polyclonal T cells including intrinsic self-reactive clones, which are silent until lymphopenia and subsequent LIP. Pathogenesis of these autoimmune diseases is consistent with the theory of “LIP-induced autoimmunity”^10^. Against expectations, transfer of highly self-reactive T cells, in which all the TCRs are specific to the recipient’s intranuclear antigens (e.g. OVA-specific T cells into intranuclear OVA-expressing recipients), did not generate LIP-Tfh and GC-B cells, suggesting that T cells with high affinity to self-antigens are bound to be silenced by anergy. Hence, the trigger of nuclear antigen-specific T cell proliferation still remains unclear. Moreover, the origin of nuclear antigen-reactive B cells, and the mechanism of their development and activation with non-self-reactive Tfh cells are yet to be elucidated.

Finally, we revealed critical roles of intestinal microbiota in lymphopenia-induced autoimmunity. By depletion of intestinal microbiota with a combination of oral antibiotics, not only LIP-Tfh cell expansion and GC formation, but also ANA production and antibody-mediated autoimmune gastritis were successfully inhibited. Recently, intensive researches into commensal microbes have revealed their roles in immune homeostasis and autoimmunity. For example, segmental filamentous bacteria induce Th17 cells in mice and are associated with exacerbation of autoimmune arthritis^37^, and clostridium species induce Treg cells in mice and humans^64^,^65^. However, specific species to induce Tfh cells have not yet been revealed. Given that autoantibody-reducing effects were altered by different combinations of oral antibiotics in our model, it is suggested that the effects are not merely attributed to the systemic effect of antibiotics but to a specific intestinal bacteria or bacterial flora. 16S rRNA gene analysis revealed that our protocol with the combination of antibiotics successfully decrease the amount and diversity of the intestinal microbiota, however, the dramatic change made it still challenging to estimate the specific microbe from the vast candidates depleted by antibiotics. It was noteworthy that pediococcus species in the family of lactobacillaceae remained after the antibiotic treatment since pediococcus and related lactic acid bacteria were reported to have probiotic effects in autoimmune diseases such as autoimmune diabetes and lupus^51^,^66^,^67^.

The place where the intestinal microbiota stimulates T cells to differentiate or to be primed into LIP-Tfh cells is also unclear. Our finding that proliferated Tc cells in spleen expressed gut homing receptor, alpha 4 beta 7 integrin, and decreased in the expression of lymphoid organ homing receptor CCR7, suggests the migration of Tc cells from lymphoid organ to the gut in the course of differentiation. Previous reports showed Tfh cells were induced by pro-inflammatory microbe in gut Peyer’s patches (PPs) and migrate to draining lymph nodes from PPs^49^. We observed PPs in Tc-transferred nude mice were almost entirely disappeared after microbiota depletion as reported previously in BALB/c WT mice^68^ (data not shown), suggesting the possible relationship of PP and LIP-Tfh cell generation.

In this study, we have also shown that CD4^+^CD25^+^ Treg cells have essential roles in the suppression of LIP-Tfh cell generation, as well as autoantibody production and autoimmune diseases^18^. Recent studies have shown that circulating Tfh-like cell populations arise and contribute to the induction of SLE-related autoantibodies in human SLE patients^50^. Several studies also identified various Treg defects in human SLE^69^. Recently a treatment to enhance in the number and function of Treg cells has been shown to succeed in a significant downregulation of Tfh cells and SLE autoantibodies as well as the improvement of SLE activity^70^. Then, microbiota-targeted strategy to induce Treg cells, in the future, might be a choice for the management of human autoimmune diseases.

Further investigation in specific microorganisms to control LIP-Tfh cells generation and LIP-associated human autoimmune diseases will pave the way to develop novel treatments for human autoimmune diseases targeting intestinal microbiota.

## Methods

### Mice

BALB/c WT and BALB/c *nu/nu* mice were obtained from Japan SLC, Inc. DO11.10 transgenic BALB/c mice whose T cells express an OVA-specific receptor were kindly provided by T. Watanabe (Medical Institute of Bioregulation, Kyushu University, Fukuoka, Japan). Rag2^−/−^ BALB/c mice and TCRα^−/−^ BALB/c mice were purchased from Taconic Farms. Scurfy C57BL/6 mice and Thy1.1^+^ BALB/c mice were obtained from the Jackson Laboratory. Ld-nOVA transgenic mice were generated as previously reported^46^, and were backcrossed to the BALB/c background for 8 generations and intercrossed, and Ld-nOVA BALB/c mice were subsequently maintained as homozygotes. Mice were bred in our facility under specific pathogen-free conditions. Female, age-matched (6–10 weeks-old at the start) mice were used in all experiments. The animal care and procedures of the experiments were approved by the Ethical Committee on Animal Experiments of the University of Tokyo, and all experiments were performed in accordance with the institutional and national guidelines.

### Cell preparation and adoptive transfer

CD4^+^ T cells were isolated from spleens using the MACS negative selection system (Miltenyi Biotec), by staining with biotinylated antibodies (Abs) against CD19 (6D5), CD8a (53–6.7), CD11b (M1/70), CD49b (DX5), I-A^b/d^ (25-9-17), and Ly-6G/Ly-6C (Gr-1, RB6-8C5) (BD Biosciences) followed by streptavidin-microbeads (Miltenyi Biotec). CD4^+^CD25^−^ cells were isolated by adding biotinylated anti-CD25 Ab (7D4) (BioLegend) to the first Abs. CD4^+^CD25^+^ cells were isolated using the MACS positive selection system by staining with PE anti-CD25 Ab followed by anti-PE microbeads (Miltenyi Biotec). Foxp3 was expressed in 96% of the CD4^+^CD25^+^ population. Purified CD4^+^ T cells, CD4^+^CD25^−^ Tc cells, or CD4^+^CD25^+^ Treg cells from WT mice were adoptively transferred into syngeneic *nu/nu* or TCRα^−/−^ mice by intraperitoneal injection (3.0 × 10^6^ cells/mice). In some experiments, donor T cells were labeled with CFSE, as previously described^7^. Briefly, cells were incubated with 5 μM CFSE for 10 min at 37 °C. B cells were positively isolated using the MACS system by staining with biotinylated anti-CD19 Ab (1D3) (BD Biosciences) followed by streptavidin-microbeads.

### Detection of antinuclear antibodies by immunofluorescence assay

Serum samples were collected via the tail vein, diluted serially from 1:40 to 1:5120 in PBS, and subjected to indirect immunofluorescence using Hep-2 cells (Fluoro HEPANA test, MBL, Nagoya, Japan) with Alexa Fluor 488-conjugated goat anti-mouse IgG (Jackson ImmunoResearch).

### ELISA for IgG, IgM and specific autoantibodies

Total IgG and IgM levels were measured using a mouse-IgG and IgM ELISA quantitation kit (Bethyl Laboratories). Ag-specific commercial ELISA kits (Orgentec) were used for the measurement of Abs against nucleosome, histone, and parietal cell. For the measurement of anti-Sm, anti-U1A, and anti-U1-68K Abs, protein antigens were obtained from Scipac. Ninety-six-well polystyrene plates were coated overnight with 50 μl of 4 μg/ml antigen in 0.03 M carbonate buffer (pH 9.6), blocked with 1% BSA and 0.05% Tween 20 in 100 μl PBS for 2 h, and incubated with serial dilutions of sera for 1 h. Then, the plates were incubated with HRP-conjugated anti-mouse IgG Abs (1:2000, Zymed Laboratories) for 30 min and visualized with 3,3′,5,5′-tetramethylbenzidine (Kirkegaard & Perry Laboratories), and the absorbance at 450 nm were measured with a plate reader (BioRad). Sera with high-titer Ab served as arbitrary units.

### Immunoprecipitation

Nuclear components were extracted from splenocytes of BALB/c WT mice using a Nuclear/Cytosol Fractionation Kit (Bio Vision), and biotinylated using a Biotinylation Kit (Sigma-Aldrich) according to the manufacturer’s directions. Protein G plus/Protein A Agarose Suspension (Calbiochem), which binds to IgG, was added to the mixture of serum and nuclear components, and incubated at 4 °C overnight. Following centrifugation and washing, bound proteins were eluted with SDS sample buffer, subjected to SDS–PAGE (on a 7.5% polyacrylamide gel), and transferred onto a PVDF membrane (Millipore Corp). Then they were incubated with HRP-conjugated streptavidin and visualized by Amersham ECL Western Blotting Detection Reagents.

### Immunization with KLH

For the induction of conventional Tfh cells, BALB/c WT mice were immunized with 200 μl of emulsion of KLH (500 μg/ml) and Complete Freund’s adjuvant injected at the base of the tail and footpad. Seven days after the immunization, inguinal and popliteal lymph nodes were analyzed.

### Flow cytometry and antibodies

For flow cytometry, lymphocytes (5 × 10^6^ cells) were incubated with fluorescence-labeled or biotinylated mAbs followed by fluorescent streptavidin, and analyzed as previously described^46^. FITC-labeled anti-T and B cell activation antigen (GL7) and anti-Foxp3 (FJK-16s) mAbs, PE-labeled anti-IL-21 (mhalx21) mAb, and PE/Cy5-labeled anti-CD4 (GK1.5) mAb were purchased from eBioscience. Biotinylated anti-PD-1 (RMP1-30), anti-CD4 (RM4-5), anti-CD25 (PC61), and anti-CD200 (OX-90) mAbs, FITC-labeled anti-ICOS (C398.4A), and anti-CD90.1 (OX-7) mAbs, PE-labeled anti-ICOS (CD278), anti-PD-1 (29F.1A12), and anti-CD200 (OX-90) mAbs, PE/Cy5 anti-CD45R/B220 (RA3-6B2), anti-CD90.2 (30-H12) mAbs, and PerCP/Cy5.5-labeled anti-CD90.1 (OX-7) mAb were purchased from BioLegend. FITC-labeled anti-CD45R/B220 (RA3-6B2), PE-labeled anti-CXCR5 (2G8), biotinylated anti-Fas (Jo2), anti-CXCR4 (2B11), anti-CD8a (53-6.7), and anti-CD19 (1D3) mAbs, and FITC and PE streptavidin were purchased from BD Biosciences. For the staining of Foxp3, a PE Anti-Mouse Foxp3 staining set (eBioscience) was used according to the manufacturer’s instructions. For the intracellular staining of IL-21, cells were incubated with lymphocyte activator cocktail (BD Biosciences) for 5 h, then incubated with intracellular fixation buffer (eBioscience) for 20 min, followed by PE-labeled anti-IL-21 mAb.

### Immunofluorescence and fluorescence microscopy

Spleens of recipient mice were frozen in OCT compound (Tissue-Tek; Sakura). Sections were cut into 7-μm slices with a microtome at −20 °C and fixed in cold acetone and then blocked with blocking buffer (Protein Block Serum-Free, Dako). Cryosections were incubated with primary antibodies, followed by staining with DyLight 488-conjugated or DyLight 594-conjugated secondary antibodies (The Jackson Laboratories).

The primary antibodies were as follows: anti-IgD (11–26c.2a, BioLegend), anti-MAdCAM-1 (MECA-367, BioLegend), anti-CD4 (GK1.5, eBioscience), and anti-PD-1 (RMP1-30, BioLegend). Rhodamine-conjugated peanut agglutinin (PNA, Vector laboratories) was used for staining of GCs. For the staining of Thy1.1, sections were incubated with biotinylated anti-CD90.1 (OX-7) mAb (AbD Serotec) followed by Streptavidin Alexa 488 (Life Technologies). Images were captured using an Olympus BX 51 fluorescent microscope equipped with an Olympus DP 71 digital camera (Olympus, Tokyo, Japan).

### RT-PCR

CD4^+^ T cells taken from recipient mice 5 days after Tc cell-transfer were separated into CD4^+^PD-1^+^ and CD4^+^PD-1^−^ cells using the MACS system. Total RNA was isolated from these purified cells and CD4^+^CD25^−^ cells from WT donors using an RNeasy Mini Kit (QIAGEN). cDNA was prepared using SuperScript TM III Reverse Transcriptase (Invitrogen) and was subjected to PCR using following primer pairs:

GAPDH-forward: 5′-GAAGGTCGGTGTGAACGGA-3′, GAPDH-reverse:5′-GTTAGTGGGGTCTCGCTCCT-3′, Bcl-6-forward: 5′-GCCCCACTGACCCGAAAGCC-3′,

Bcl-6-reverse:5′-GCCTGCCAGGGACCTGTTCAC-3′, IL-21-forward:5′-ATCCTGAACTTCTATCAGCTCCAC-3′, IL-21-reverse: 5′-GCATTTAGCTATGTGCTTCTGTTC-3′.

### Co-culture of CD4^+^ T cells and B cells

CD4^+^CD25^−^ Tc cells and CD19^+^ B cells were isolated from WT mice, and CD4^+^PD-1^+^ or CD4^+^PD-1^−^ T cells were isolated from *nu/nu* mice 5 days after Tc cell-transfer using the MACS system. CD19^+^ B cells (2 × 10^5^/well) and subsets of CD4^+^ T cells (2 × 10^4^/well) were co-cultured in 96-well round-bottomed plates in RPMI 1640 medium containing 10% FCS at 37 °C with 5% CO_2_. CD4^+^ T cells were stimulated with 10 μg/ml of immobilized anti-CD3 mAb and 5 μg/ml of anti-CD28 mAb (BD Pharmingen). In some experiments, 5 μg/ml of anti-ICOS mAb (7E.17G9, BioLegend) and/or 10 μg/ml of anti-IL-21 Ab (R&D systems) were added to the culture. After 96 h, the concentration of total IgG in supernatant was measured by ELISA.

### Histopathological assessment

Colons and stomachs were excised at necropsy. Tissues were fixed with 10% neutral formalin, paraffin embedded, sectioned at 3–6 μm, and stained with hematoxylin and eosin. Colitis was defined by the presence of cell infiltration, loss of goblet cells, mucin depletion, crypt elongation, and crypt abscesses. Gastritis was defined by the presence of cell infiltration, oxyntic atrophy, and hyperplasia/metaplasia. Images were captured using an Olympus BX 51 microscope equipped with an Olympus DP 71 digital camera.

### Antibiotics treatment

To deplete intestinal microbiota in recipient *nu/nu* mice, mice were treated with a combination of antibiotics according to previous protocols with minor modifications^48^. Ciprofloxacin (250 mg/l; Sigma-Aldrich), imipenem/cilastatin (250 mg/l; MSD), metronidazole (250 mg/l; Sigma-Aldrich), vancomycin (250 mg/l; Sigma-Aldrich), and amphotericin B (5 mg/l; Bristol-Myers Squibb) were added to the drinking water of *nu/nu* mice and the mice were allowed to drink water ad libitum. Administration was started 5 days prior to T cell transfer and was continued during experiments. Water was changed every 3 days.

### 16S rDNA acquisition from fecal samples and semi-quantification of 16S rDNA

Stool samples were collected from mice before, 2 weeks after, or 4 weeks after antibiotic treatment with CPFX, IPM, MDZ, and VCM and frozen and stored at −80 °C. DNA extraction was done as described previously with some modifications^71^. The stool samples were homogenized in TE buffer and treated with Achromopeptidase and Lysozyme at 37˚C for an hour. DNA was purified with phenol/chloroform extraction and isopropanol precipitation. 16S rRNA gene fragment including V3 and V4 region were amplified by PCR (forward primer: ACACGACGCTCTTCCGATCTCCTACGGGNGGCWGCAG, reverse primer: GACGTGTGCTCTTCCGATCTGACTACHVGGGTATCTAATCC, underline: overhang sequence for 2nd PCR) for 20 cycles, and PCR products were purified with Agencourt AMpure beads (Beckman coulter). Then, overhang and index sequences for sequencing were added by 2nd PCR with NEBNext multiplex Oligos for Illumina (Dual Index Primers Set1, New England Biolabs) for 8 cycles and the products were purified with Agencourt AMpure beads.

16S V3-V4 rDNA in the stool DNA sample, normalized with stool amount (40 μg), were amplified by PCR with the 1st primers for 24 cycles. Then agarose gel electrophoresis was performed and 16S rDNA product bands were semi-quantified by ImageJ (NIH software).

### 16S rDNA sequence data analysis

Raw Illumina MiSeq 2 × 300 paired-end reads were quality filtered with the following steps: (1) adaptor sequence trimming, (2) low-quality base trimming and (3) pair-end read overlapping. Firstly, adaptor sequences were removed by cutadapt (v.1.2.1)^72^. In the second step, the first and last 10 nucleotides from each read were removed, low quality nucleotides with quality score less than 20 and nucleotides in between from both ends searched with widow size 10 were trimmed, and trimmed reads shorter than 75 were discarded. After the trimming, low complexity sequences, sequences containing N at more than 1% and singletons were filtered out. This step was performed by PRINSEQ (lite v.0.20.4)^73^ (-trim_right 10 -trim_left 10 -trim_qual_right 20 -trim_qual_left 20 -trim_qual_window 10 -min_len 75 −lc_method dust −lc_threshold 7 -ns_max_p 1). Finally, the reads of each pair were aligned and the optimal overlap was determined and form a single assembled read by PEAR (v.0.9.6)^74^ (default settings). The assembled reads were used for further analyses.

16S analysis was performed with QIIME (Quantitative Insights Into Microbial Ecology, version 1.9.1)^75^. The quality filtered reads were assigned to closed reference operational taxonomic units (OTUs) at a 97% identity threshold using the Greengenes database (version 13.8)^76^. To account for inter-sample sequencing depth variability, all samples were rarefied to 15,000 reads per sample (10 iterations). Rarefaction curves of observed OTUs were calculated using QIIME. Two-tailed unpaired Student’s t test was used to compare between the groups.

### Statistics

Statistical analysis was performed using two-tailed unpaired Student’s *t*-test. *P* values < 0.05 were considered significant unless stated otherwise. All error bars represent the SEM.

## Additional Information

**How to cite this article**: Eri, T. *et al*. Intestinal microbiota link lymphopenia to murine autoimmunity via PD-1+CXCR5^−/dim^ B-helper T cell induction. *Sci. Rep.* **7**, 46037; doi: 10.1038/srep46037 (2017).

**Publisher's note:** Springer Nature remains neutral with regard to jurisdictional claims in published maps and institutional affiliations.

## Supplementary Material

Supplementary Information
