# Supplementary material for: Intestinal microbiota link lymphopenia to murine autoimmunity via PD-1+CXCR5−/dim B-helper T cell induction
Source: Sci Rep. 2017 Apr 26;7:46037. doi: 10.1038/srep46037 (PMC5405410; doi:10.1038/srep46037)
Supplement: Supplementary Information [file srep46037-s1.pdf]

Supplementary Information

Intestinal microbiota link lymphopenia to murine autoimmunity via PD-1^{+}CXCR5$^{-/dim}$ B-helper T cell induction

Toshiki Eri, Kimito Kawahata, Takeyuki Kanzaki, Mitsuru Imamura, Kazuya Michishita, Lisa Akahira, Ei Bannai, Noritada Yoshikawa, Yasumasa Kimura, Takeshi Satoh, Satoshi Uematsu, Hirotoshi Tanaka & Kazuhiko Yamamoto

Supplementary Figure S1 related to Fig. 2

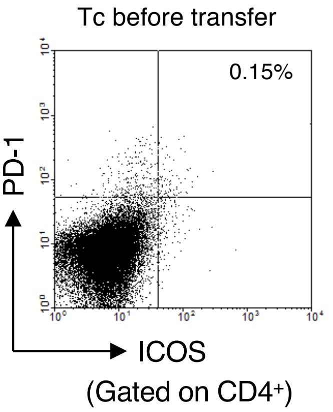

Figure S1: PD-1^{+}ICOS^{+}CD4^{+} Tfh cells are very few among Tc cells before transfer.
Flow cytometry analysis for PD-1 and ICOS on CD4^{+}CD25^{-} Tc cells derived from spleen of WT mice. Tc cells are isolated by MACS negative selection. The number in the plot represents the percentage of PD-1^{+}ICOS^{+} cells.

Supplementary Figure S2

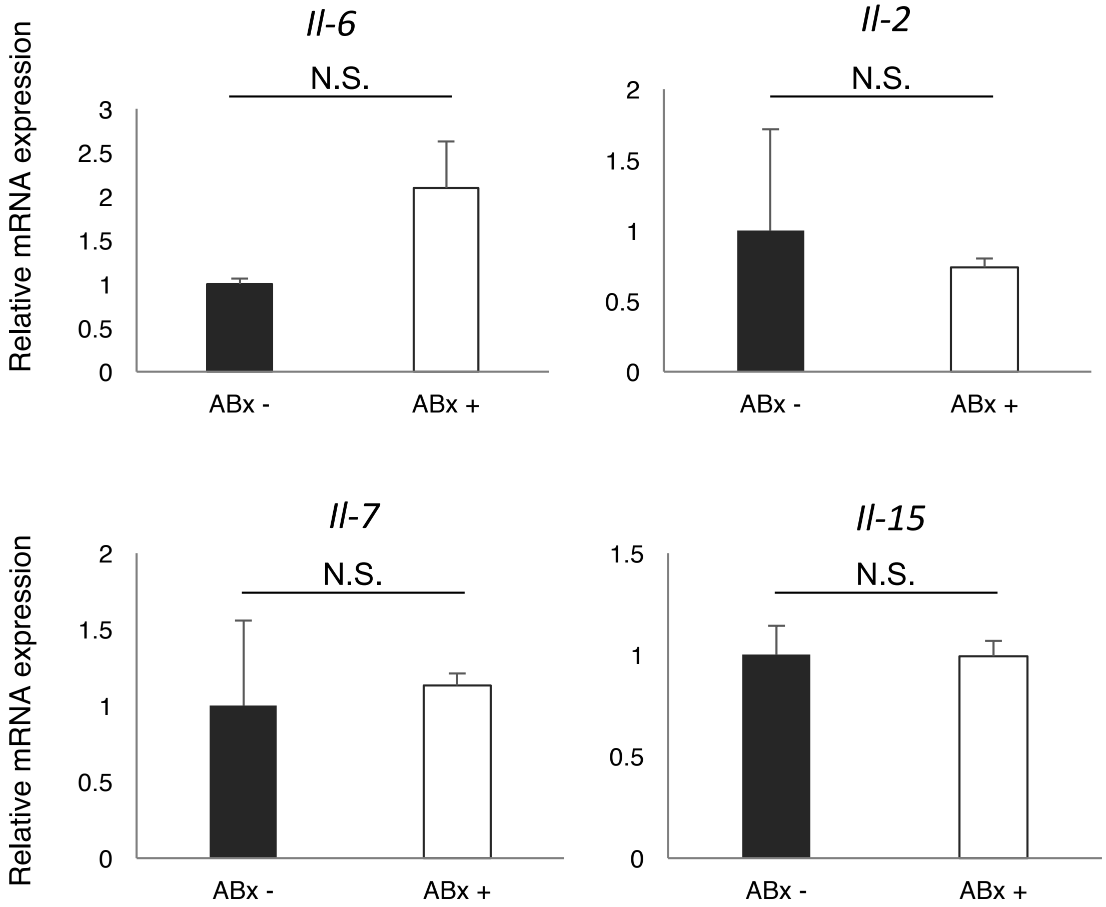

Figure S2: Cytokine profile in the spleen of *nu/nu* mice after antibiotic treatment.
mRNA expression of IL-6, IL-2, IL-7, and IL-15 in splenocytes from control *nu/nu* mice (ABx-) and antibiotic-treated *nu/nu* mice (ABx+) were quantified by real-time quantitative RT-PCR.
n = 3 per group per cytokine, mean ± SEM. N.S., not significant.

Supplementary Figure S3 related to Fig. 7

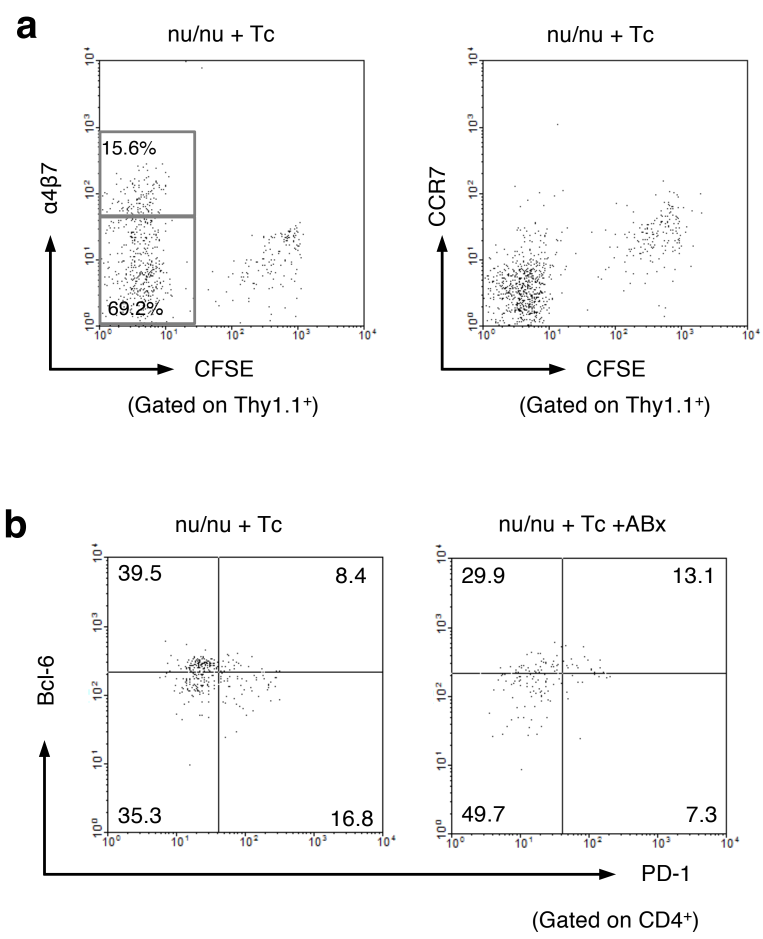

Figure S3: Relationship between LIP-Tfh differentiation and intestinal microbiota.
(a) CFSE-stained Thy1.1$^+$ Tc cells were transferred into nu/nu mice. Expression of alpha 4 beta 7 integrin and CCR7 on the transferred and proliferated cells were analyzed by FACS. (b) Intracellular Bcl-6 expression in PD-1$^+$ LIP-Tfh cells were analyzed by flow cytometry. *Nu/nu* mice were treated with oral combination of antibiotics (ABx) (CPFX, IPM, MDZ, and VCM) in drinking water for 10 days, then Tc cells from WT mice were transferred i.p.. Splenocytes were taken from recipient mice 5 days after the transfer. Intracellular staining of Bcl-6 was performed after staining surface markers and following intracellular fixation and permeabilization.
Data plots were representative of 2 independent experiments with 3 mice per group. Numbers in the plot represent the percentage of gated cells.

Supplementary Experiment Method

Quantitative RT-PCR analysis of cytokines

Total RNA was extracted from crushed frozen spleen tissues from antibiotic-treated or non-treated mice using Sepasol-RNA I Super G (Nacalai tesque) and subjected to reverse-transcription with oligo-dT primer (Invitrogen) and ReverTra Ace MMLV Reverse Transcriptase (TOYOBO). Real-time PCR was performed with THUNDERBIRD Probe qPCR Mix (TOYOBO), Universal ProbeLibrary Sets (Roche) and CFX96 Real-Time PCR Detection System (Bio-Rad). Expression levels of mRNA were calculated on the basis of standard curves generated for each primer pair and beta-actin mRNA was used as an invariant control. Sequences of primers used in qRT-PCR are listed below:

IL-2: 5’-gctgttgatggacctacagga-3’ and 5’-atcctggggagtttcaggtt-3’
IL-6: 5’-gctaccaaactggatataatcagga-3’ and 5’-ccaggtagctatggtactccagaa-3’
IL-7: 5’-cgcagaccatgttccatgt-3’ and 5’-tctttaatgtggcactcagatgat-3’
IL-15: 5’-cagaggccaactggatagatg-3’ and 5’-actgtcagtgtataaagtggtgtcaat-3’
Beta-actin: 5’-aaggccaaccgtgaaaagat-3’ and 5’-gtggtacgaccagaggcatac-3’
